# Integrated evaluation of rapid diagnostic testing, genotypic-phenotypic resistance profiling, and AI-Assisted prediction models for antimicrobial stewardship and clinical outcomes in a resource-limited setting

**DOI:** 10.1371/journal.pone.0347223

**Published:** 2026-08-20

**Authors:** Muhammad Hammad, Rasikh Arif, Sadaf Fardoos, Khadija Shakoor, Ali Nasir

**Affiliations:** 1 Department of Community Health Sciences, Sohail University, Karachi, Pakistan; 2 Shifa College of Pharmaceutical Sciences, Shifa Tameer-e- Millat University, Islamabad, Pakistan; 3 School of Public Health, AI-Shifa Eye Trust Hospital, Rawalpindi, Pakistan; 4 Department of Pharmaceutical Sciences, Riphah International University, Islamabad, Pakistan; 5 MBBS Student, HBS Medical and Dental College, Islamabad, Pakistan; 6 Department of Pharmacology, Ziauddin University & Hospital, Karachi, Pakistan; Yale University School of Medicine, UNITED STATES OF AMERICA

## Abstract

**Introduction:**

Antimicrobial resistance (AMR) remains a major global health concern, necessitating timely and accurate diagnostic approaches to guide appropriate therapy. Conventional antibiotic susceptibility testing (AST) is often associated with delays that may compromise clinical outcomes.

**Objective:**

To evaluate the impact of integrating rapid diagnostic testing, genotypic resistance profiling, and artificial intelligence (AI)-based prediction models on antimicrobial stewardship and clinical outcomes.

**Methods:**

A prospective observational study was conducted at Lady Reading Hospital, MTI, Peshawar, Pakistan from 13/02/2023–18/04/2025, including 410 adult patients with suspected bacterial infections. Participants underwent conventional AST, rapid diagnostic testing, and molecular detection of resistance genes. An AI-based model was developed using clinical and laboratory parameters to predict antimicrobial resistance. Key outcomes included time to effective therapy, antibiotic appropriateness, length of hospital stay, and mortality. Statistical analysis included comparative tests, logistic regression, and receiver operating characteristic (ROC) curve analysis and was performed using SPSS Version 26.0 and R version 4.5.2.

**Results:**

Rapid diagnostics significantly reduced time to pathogen identification (10.4 vs 48.6 hours, p < 0.001) and initiation of effective therapy (20.3 vs 55.8 hours, p < 0.001). Appropriate antibiotic use improved from 54.2% to 78.3% (p < 0.001), while broad-spectrum antibiotic use declined significantly. The integrated approach was associated with reduced hospital stay (8.6 vs 12.9 days, p < 0.001) and lower mortality (14.0% vs 22.7%, p = 0.02). Genotypic–phenotypic concordance was substantial (κ = 0.61–0.69). The AI model demonstrated strong predictive performance (AUC = 0.89).

**Conclusion:**

An integrated diagnostic and predictive approach was associated with improvements in antimicrobial stewardship and favorable clinical outcomes, suggesting its potential as a cost-effective and scalable strategy for addressing antimicrobial resistance (AMR), particularly in resource-limited settings.

## Introduction

Antimicrobial resistance (AMR) has emerged as one of the most significant global public health threats, compromising the effectiveness of antimicrobial therapies and contributing to increased morbidity, mortality, prolonged hospitalization, and healthcare expenditure [1]. Timely and accurate identification of resistant pathogens is central to effective clinical management, as it enables clinicians to initiate targeted antimicrobial therapy and optimize patient outcomes while minimizing unnecessary exposure to broad-spectrum antibiotics [2]. Conventional antibiotic susceptibility testing (AST), although regarded as the reference standard for determining antimicrobial susceptibility, typically requires 48–72 hours to generate actionable results. This delay frequently necessitates empirical broad-spectrum antibiotic use, which may contribute to inappropriate antimicrobial exposure and further acceleration of resistance development [3].

Recent advances in rapid diagnostic technologies have substantially improved the ability to identify pathogens and detect resistance-associated markers within shorter timeframes [4]. Molecular diagnostic platforms, including multiplex polymerase chain reaction (PCR)-based assays and rapid microbial identification systems, facilitate early detection of clinically important resistance genes and may support earlier optimization of antimicrobial therapy [5]. However, discordance between genotypic resistance markers and phenotypic susceptibility patterns has been increasingly recognized, particularly in organisms with complex or multifactorial resistance mechanisms. Consequently, understanding the concordance and limitations of genotypic and phenotypic methods remains important for appropriate interpretation of diagnostic results and clinical decision-making [3].

In parallel, artificial intelligence (AI) and machine learning approaches are increasingly being explored in clinical microbiology and infectious disease management as adjunctive tools for predicting antimicrobial resistance using routinely available demographic, clinical, laboratory, and microbiological data [6]. Previous studies have demonstrated promising predictive performance of machine learning models for forecasting resistance patterns and supporting antimicrobial selection [7]. Nevertheless, most published models remain derivation-based and require external validation prior to widespread clinical implementation. Moreover, model calibration, generalizability, and integration into real-time clinical workflows remain ongoing challenges, particularly in low- and middle-income healthcare settings.

Antimicrobial Stewardship Programs (ASPs) play a critical role in operationalizing diagnostic and predictive tools by integrating microbiological data into evidence-based antimicrobial decision-making [8]. Rapid diagnostics, molecular resistance profiling, and predictive algorithms may enhance stewardship interventions by facilitating earlier escalation, de-escalation, or optimization of antibiotic therapy. In resource-constrained healthcare systems, where delays in microbiological reporting and limited stewardship infrastructure are common, integrated diagnostic approaches may improve antimicrobial utilization while supporting more efficient allocation of healthcare resources.

Despite these advances, important evidence gaps remain regarding the combined clinical utility of rapid diagnostic testing, genotypic resistance profiling, and AI-assisted prediction models within real-world clinical workflows, particularly in low- and middle-income countries [9]. Most previous studies have evaluated rapid diagnostics, genotypic resistance profiling, and artificial intelligence-based prediction approaches as distinct components, with comparatively limited evidence on their integrated application within routine clinical workflows and their combined association with antimicrobial stewardship outcomes and patient-centered clinical endpoints [10,11]. Furthermore, evidence regarding the feasibility, operational integration, and implementation of such multimodal approaches in resource-limited healthcare environments remains limited, with challenges related to infrastructure, resourcing, logistics, and integration into antimicrobial stewardship workflows [12].

Therefore, this prospective observational cohort study aimed to evaluate the association between an integrated diagnostic stewardship approach incorporating rapid diagnostic testing, genotypic resistance profiling, and machine learning-based prediction models and key antimicrobial stewardship metrics and clinical outcomes among adult patients with suspected bacterial infections. The primary estimand was the between-group difference in the mean-time from clinical suspicion of bacterial infection to initiation of effective antimicrobial therapy among patients managed using rapid diagnostic–supported workflows compared with those managed using conventional diagnostic workflows. Secondary outcomes included time to pathogen identification, time to antimicrobial susceptibility reporting, appropriateness of antimicrobial therapy, rates of antibiotic escalation and de-escalation, length of hospital stay, ICU admission, and in-hospital mortality. In addition, the study assessed the concordance between genotypic and phenotypic antimicrobial resistance profiles and evaluated the discriminatory performance and calibration of machine learning-based prediction models for identifying antimicrobial resistance and assessing their potential utility as adjunctive tools for antimicrobial decision-making.

## Materials and methods

### Study design

This prospective observational cohort study was conducted to examine the association between an integrated diagnostic stewardship strategy and antimicrobial stewardship outcomes among adult patients with suspected bacterial infections. The integrated approach incorporated rapid diagnostic testing, genotypic resistance profiling, and machine learning–based antimicrobial resistance prediction models. The prospective design enabled evaluation of diagnostic pathways and their association with antimicrobial prescribing practices in routine clinical settings. Participants were followed from the initial clinical suspicion of bacterial infection through microbiological confirmation, treatment modification, and final in-hospital outcomes.

The primary outcome was the time to initiation of effective antimicrobial therapy, defined as the interval between clinical suspicion of bacterial infection and administration of an antimicrobial agent subsequently confirmed to be microbiologically appropriate based on phenotypic antimicrobial susceptibility testing (AST). The primary estimand was the between-group difference in mean time to effective antimicrobial therapy between patients managed using rapid diagnostic–supported workflows and those managed using conventional diagnostic workflows. Secondary outcomes included time to pathogen identification, time to antimicrobial susceptibility reporting, time to antibiotic modification, appropriateness of antimicrobial therapy, rates of antibiotic escalation and de-escalation, length of hospital stay, ICU admission, and in-hospital mortality. Additional analyses assessed diagnostic turnaround times for rapid diagnostic platforms compared with conventional AST, concordance between genotypic resistance markers and phenotypic susceptibility results, and the discriminatory and predictive performance of machine learning models for antimicrobial resistance prediction and assessment of antimicrobial appropriateness.

Patients were categorized according to the diagnostic workflow available during their clinical management. Those managed during the pre-implementation period, when conventional diagnostic workflows were used, constituted the conventional diagnostic group, whereas those managed during the post-implementation period, when rapid diagnostic–supported workflows were available, constituted the rapid diagnostic group. Group assignment was determined by the implementation period and availability of the respective diagnostic workflow during routine clinical care and was not randomized. All patients underwent conventional phenotypic AST as the reference method, while rapid diagnostic testing was additionally available to patients managed during the post-implementation period.

The study was reported in accordance with the Strengthening the Reporting of Observational Studies in Epidemiology (STROBE) guidelines, with the development and reporting of the machine learning prediction model additionally guided by relevant principles of the Transparent Reporting of a Multivariable Prediction Model for Individual Prognosis or Diagnosis (TRIPOD) guidelines.

### Sample size

Sample size was calculated using a single population proportion formula based on an anticipated antimicrobial resistance prevalence of 58.2% [13], based on previously published data. With a 95% confidence level and a margin of error of 5%, the minimum required sample size was estimated to be 374 participants. To account for potential data loss, incomplete records, or dropouts, an additional 10% was added, resulting in a final target sample size of approximately 410 patients. The sample size was determined primarily based on the anticipated prevalence of antimicrobial resistance and the specified precision of the prevalence estimate. The available sample was subsequently used for comparative analyses of diagnostic and clinical outcomes, multivariable regression analyses, and exploratory machine learning model development. For machine learning model development, internal validation procedures including cross-validation and regularization techniques were implemented to minimize overfitting risk. However, the prediction model should be considered a derivation model requiring future external validation prior to routine clinical implementation.

### Study setting

Study was carried out at Lady Reading Hospital, MTI, Peshawar, Pakistan, a tertiary care teaching hospital, incorporating the Departments of Internal Medicine, Intensive Care Units (ICUs), and the Clinical Microbiology Laboratory. Study was conducted in accordance with the principles of the Declaration of Helsinki (64th WMA General Assembly, Fortaleza, Brazil, 2013). Protocol approval was obtained from Ethical Review Committee of Lady Reading Hospital, MTI, Peshawar, Pakistan (Reference No: 605/LRH/MTI, Date: 04/02/2023). Written informed consent was obtained from all participants or their legally authorized representatives before enrollment. The microbiology laboratory is well-equipped with conventional culture systems, automated microbial identification platforms, and standardized antibiotic susceptibility testing (AST) methods, along with molecular diagnostic facilities for detecting resistance genes. This integrated infrastructure enabled a simultaneous comparison of conventional phenotypic methods, rapid diagnostic platforms, and genotypic resistance profiling within routine clinical workflows. Inclusion of both ICU and non-ICU settings ensured representation of diverse infection severities, including community-acquired and healthcare-associated infections, thereby enhancing the generalizability of the findings.

### Study duration

Study was conducted from 13/02/2023–18/04/2025, which allowed adequate capture of seasonal variations in infection patterns and antimicrobial resistance trends. An additional 4–6 months were allocated for data cleaning, validation, statistical analysis, and development and evaluation of AI-based predictive models, ensuring methodological rigor and completeness of the study.

### Study population (Inclusion and Exclusion Criteria)

Adult patients aged 18 years or older presenting with clinically suspected bacterial infections, including bloodstream infections, respiratory tract infections, and urinary tract infections, were considered eligible for inclusion. Patients were included only if conventional phenotypic antibiotic susceptibility testing, rapid diagnostic testing, and corresponding clinical management data were available for analysis. Patients were excluded if they had received systemic antibiotic therapy for more than 48 hours prior to presentation, as prior antimicrobial exposure could alter microbiological yield and resistance profiles. Moreover, exclusion criteria included incomplete clinical or microbiological records, refusal to provide informed consent, and confirmed non-bacterial infections such as viral, fungal, or parasitic etiologies. Polymicrobial infections were excluded because attribution of phenotypic resistance patterns and antibiotic appropriateness to individual organisms could not be reliably determined, potentially introducing diagnostic and therapeutic misclassification bias.

### Study outcomes

Primary outcomes of the study included time to initiation of effective antibiotic therapy and the appropriateness of antimicrobial treatment based on microbiological susceptibility results. Secondary outcomes included in-hospital mortality, length of hospital stays, ICU admission rates, and rates of antibiotic escalation and de-escalation. Moreover, the study evaluated the concordance between genotypic and phenotypic resistance patterns and assessed the predictive performance of AI-based models in identifying antimicrobial resistance and guiding appropriate therapy. These outcomes were selected to comprehensively assess both clinical effectiveness and stewardship impact.

### Diagnostic and laboratory procedures

**Fig 1:**
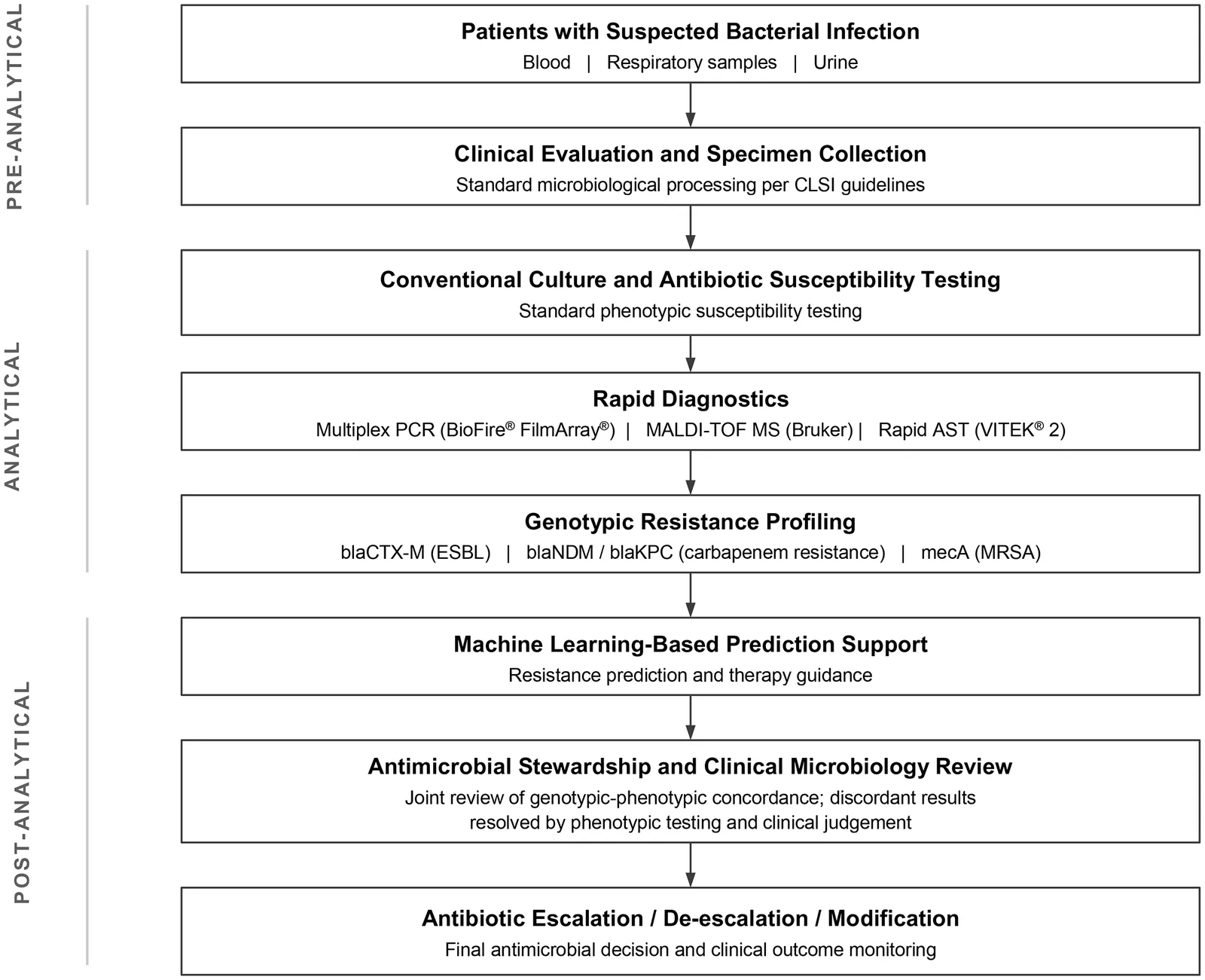
Integrated Diagnostic and Antimicrobial Stewardship Workflow Algorithm.

All clinical specimens, including blood, respiratory samples, and urine, were processed using standard microbiological techniques. Conventional culture and antibiotic susceptibility testing were performed in accordance with established laboratory protocols and Clinical and Laboratory Standards Institute (CLSI) guidelines [14]. Rapid diagnostic testing platforms, including multiplex real-time PCR (BioFire® FilmArray®, BioFire Diagnostics, Salt Lake City, UT, USA), MALDI-TOF mass spectrometry (Bruker Daltonics, Bremen, Germany), and automated antimicrobial susceptibility testing (AST) platforms (VITEK® 2, bioMérieux, Marcy-l’Étoile, France), were utilized for early pathogen identification and antimicrobial susceptibility assessment. These platforms provided earlier microbiological information than conventional culture-based workflows, and their association with diagnostic turnaround times was evaluated. Molecular diagnostic assays were employed for genotypic resistance profiling, targeting clinically relevant resistance genes such as blaCTX-M for extended-spectrum beta-lactamases, blaNDM and blaKPC for carbapenem resistance, and mecA for methicillin-resistant *Staphylococcus aureus* (MRSA). Discordant genotypic-phenotypic results were reviewed jointly by the clinical microbiology and antimicrobial stewardship teams. In cases where molecular resistance markers were detected without corresponding phenotypic resistance, final antimicrobial decisions were guided primarily by phenotypic AST findings and clinical judgment. Moreover, confirmatory testing was performed where clinically indicated, and discordant results were not used as sole determinants for antimicrobial escalation. The integrated diagnostic and antimicrobial stewardship workflow is shown in (Fig 1).

### Antimicrobial Stewardship workflow

An institutional antimicrobial stewardship framework was utilized throughout the study period. Initial empirical antimicrobial therapy was selected according to local institutional guidelines and clinician judgment at the time of presentation. Rapid diagnostic and molecular resistance results were subsequently communicated to treating clinicians and antimicrobial stewardship personnel to facilitate reassessment of antimicrobial therapy. Decisions regarding antibiotic escalation, de-escalation, continuation, or modification were made through integration of clinical status, rapid diagnostic findings, genotypic resistance markers, and final phenotypic AST results. Machine learning model outputs were evaluated as adjunctive clinical decision-support tools and were not independently used to direct antimicrobial prescribing. Final treatment decisions remained clinician-directed throughout the study period.

### Machine learning prediction model

A machine learning-based prediction model was developed to estimate the probability of antimicrobial resistance and likelihood of appropriate antimicrobial therapy. Input variables included age, sex, diabetes mellitus, hypertension, chronic kidney disease, source of infection, ICU status at initial presentation, prior antimicrobial exposure, white blood cell count, neutrophil count, C-reactive protein, procalcitonin, serum creatinine, and preliminary microbiological findings available at or shortly after initial clinical presentation. To preserve temporal validity and minimize information leakage, only variables available at or shortly after initial clinical presentation and before the initial antimicrobial modification were included in model development; post-outcome information was not incorporated. The dataset was randomly divided into training and validation subsets using a 70:30 ratio. Model development and hyperparameter tuning were performed exclusively within the training dataset using stratified 5-fold cross-validation, while the 30% validation dataset was held out from model training and hyperparameter optimization and was used only for final internal performance assessment. This approach was used to reduce optimism in performance estimates and minimize overfitting. Missing data were managed using multiple imputation by chained equations (MICE) to minimize bias associated with incomplete observations. Overall missingness for variables included in predictive modeling was below 10%. Variables with missingness exceeding 20% were excluded from predictive modeling analyses to minimize instability and potential imputation bias. Logistic regression with L2 regularization and random forest ensemble algorithms were implemented for predictive modeling. Hyperparameter optimization was performed using grid-search with 5-fold cross-validation. Class imbalance in antimicrobial resistance outcomes was assessed during model development, and stratified cross-validation procedures were utilized where appropriate to maintain balanced representation across training and validation subsets. Feature importance was evaluated using standardized regression coefficients for logistic regression and Gini impurity measures for random forest models. Model performance assessment included both discrimination and calibration analyses. Discriminatory performance was evaluated using area under the receiver operating characteristic curve (AUC), sensitivity, specificity, positive predictive value (PPV), negative predictive value (NPV), and overall accuracy. Calibration assessment was additionally performed using calibration plots and Hosmer-Lemeshow goodness-of-fit testing. The prediction model underwent internal validation only and should therefore be interpreted as a derivation model requiring future external validation prior to routine clinical implementation. The machine learning model was developed and evaluated as an adjunctive prediction tool. Model predictions were generated for eligible patients based on variables available at or shortly after initial clinical presentation. During the study workflow, model outputs were available to the clinical research and antimicrobial stewardship team for evaluation but were not used as an autonomous prescribing mechanism. Antibiotic decisions remained under the responsibility of treating clinicians and were based on clinical assessment, microbiological findings, rapid diagnostic results, genotypic resistance markers, phenotypic AST, and, where applicable, the model-predicted probability of resistance.

### Cost comparison analysis

A comparative cost analysis was conducted from the hospital perspective to compare direct healthcare costs associated with conventional and rapid diagnostic workflows during the index hospitalization. The analysis included diagnostic testing costs and hospitalization-related costs incurred during the index admission.

Diagnostic costs were estimated using the institutional charges applicable to conventional microbiological testing and rapid diagnostic procedures, respectively. The diagnostic cost estimates were PKR 12,600 per patient for conventional diagnostic testing and PKR 33,600 per patient for rapid diagnostic testing.

Hospitalization costs were estimated using patient-level inpatient cost data available from the institutional records for the index hospitalization. The estimated hospitalization cost was derived from patient-level inpatient cost data recorded in the institutional financial records for the index hospitalization and incorporated inpatient resource utilization and the institutional cost structure. Where applicable, differences between ward-based and ICU-based care were incorporated into the hospitalization cost estimates. The hospitalization cost estimates therefore reflected the average inpatient costs observed for patients managed under the respective diagnostic workflows rather than a simple multiplication of mean length of stay by a single uniform daily rate.

The average hospitalization costs were PKR 406,000 per patient in the conventional diagnostic group and PKR 274,400 per patient in the rapid diagnostic group. The mean total cost per patient was calculated as the sum of the mean diagnostic testing cost and mean hospitalization cost. Accordingly, the estimated mean total cost was PKR 418,600 per patient in the conventional diagnostic group and PKR 308,000 per patient in the rapid diagnostic group, corresponding to an estimated difference of PKR 110,600 per patient.

The analysis was conducted from the hospital perspective and included costs incurred during the index hospitalization only. Indirect costs, productivity losses, outpatient costs, post-discharge healthcare utilization, and long-term costs were not included. No discounting or formal sensitivity analysis was performed. Therefore, the findings are presented as a comparative cost analysis rather than a formal cost-effectiveness or cost-utility analysis.

### Data collection procedures

Data were collected using a structured and standardized case report form designed specifically for the study. Information collected included demographic characteristics, comorbid conditions, clinical presentation, type of infection, initial empirical antibiotic therapy, microbiological findings, molecular resistance profiles, and subsequent treatment modifications. Patients were followed throughout their hospital stay to document outcomes such as time to effective therapy, duration of hospitalization, ICU admission, and mortality. Data entry was performed using a secure electronic database with built-in validation checks to minimize errors. Regular data audits and quality control procedures were implemented to ensure accuracy and completeness.

### Statistical analysis

All statistical analyses were conducted using SPSS Version 26.0 and R version 4.5.2 statistical software. Descriptive statistics were used to summarize baseline characteristics, microbiological profiles, and resistance patterns. Continuous variables were expressed as mean ± standard deviation or median with interquartile range, depending on data distribution, while categorical variables were presented as frequencies and percentages. Comparative analyses between groups were performed using independent t-tests or Mann-Whitney U tests for continuous variables and chi-square or Fisher’s exact tests for categorical variables. Agreement between genotypic and phenotypic resistance was assessed using Cohen’s kappa coefficient. Multivariable logistic regression models were constructed based on clinically relevant covariates and previously established predictors identified from published literature rather than solely on statistical significance testing, thereby minimizing inappropriate data-driven variable selection bias. Clinically important variables were retained a priori in adjusted analyses irrespective of univariable statistical significance. Adjusted effect estimates were reported as odds ratios (ORs), adjusted odds ratios (AORs), 95% confidence intervals (CIs), and p-values. Machine learning model performance was evaluated using ROC analysis, calibration assessment, and classification performance metrics. A two-sided p-value <0.05 was considered statistically significant.

## Results

A total of 410 patients with clinically suspected bacterial infections were included in the final analysis. The mean age of participants was 52.6 ± 17.4 years, reflecting a predominantly middle-aged to elderly cohort. The largest proportion of patients belonged to the 41–60 years age group (39.5%), followed by patients aged >60 years (31.7%) and 18–40 years (28.8%). Male participants comprised 58.0% of the study population. Diabetes mellitus (38.0%) and hypertension (34.6%) were the most common comorbid conditions, while bloodstream infections represented the most frequent infection type (41.0%). Approximately one-third of patients (35.6%) required ICU admission during hospitalization. Baseline demographic and clinical characteristics are summarized in Table 1.

**Table 1:** Demographic, Clinical, and Infection-Related Characteristics of the Study Participants (n = 410.

Variable	Category	Frequency (n)	Percentage (%)
Age (years)	Mean ± SD	52.6 ± 17.4	
Age Group	18-40	118	28.8
	41-60	162	39.5
	>60	130	31.7
Gender	Male	238	58.0
	Female	172	42.0
Comorbidities	Diabetes Mellitus	156	38.0
	Hypertension	142	34.6
	CKD	64	15.6
	None	96	23.4
Type of Infection	Bloodstream Infection	168	41.0
	Respiratory Infection	124	30.2
	UTI	98	23.9
	Others	20	4.9
ICU Admission	Yes	146	35.6
	No	264	64.4

Table 2 summarizes the microbiological profile of isolates identified in the study population. A predominance of Gram-negative organisms was observed, accounting for 67.8% of all isolates, while Gram-positive bacteria comprised 32.2%. Among the specific pathogens, *Escherichia coli* was the most frequently isolated organism (28.8%), followed by *Klebsiella* species (22.4%) and *Staphylococcus aureus* (23.4%). *Pseudomonas aeruginosa* accounted for 9.8% of isolates, whereas other identified bacterial organisms collectively accounted for 15.6% of the study isolates.

**Table 2:** Distribution of Bacterial Isolates Identified Among the Study Participants.

Organism	Frequency (n)	Percentage (%)
Gram-negative bacteria	278	67.8
Gram-positive bacteria	132	32.2
*E. coli*	118	28.8
*Klebsiella spp.*	92	22.4
*Pseudomonas aeruginosa*	40	9.8
*Staphylococcus aureus*	96	23.4
Others	64	15.6

Rapid diagnostic platforms demonstrated significantly shorter turnaround times compared with conventional antibiotic susceptibility testing (AST). Mean time to pathogen identification was reduced from 48.6 ± 8.5 hours with conventional AST to 10.4 ± 3.2 hours using rapid diagnostics (mean difference: −38.2 hours; 95% CI: −39.3 to −37.1; p < 0.001). Similarly, time to susceptibility reporting was substantially shorter in the rapid diagnostics group (14.8 ± 4.1 hours versus 60.2 ± 10.3 hours; mean difference: −45.4 hours; 95% CI: −46.8 to −44.0; p < 0.001). Time to antibiotic modification was also significantly reduced among patients evaluated using rapid diagnostic approaches (18.6 ± 6.2 hours versus 52.4 ± 9.7 hours; mean difference: −33.8 hours; 95% CI: −35.1 to −32.5; p < 0.001). Diagnostic accuracy of rapid diagnostic platforms compared with conventional phenotypic AST was 92.3%. Detailed comparative findings are presented in Table 3.

**Table 3:** Comparison of Diagnostic Turnaround Times Between Rapid Diagnostic-Supported and Conventional Diagnostic Workflows.

Parameter	Rapid Diagnostics	Conventional AST	Mean Difference / Effect Estimate	p-value
Time to identification (hours)	10.4 ± 3.2	48.6 ± 8.5	−38.2 (95% CI: −39.3 to −37.1)	<0.001
Time to susceptibility result (hours)	14.8 ± 4.1	60.2 ± 10.3	−45.4 (95% CI: −46.8 to −44.0)	<0.001
Time to antibiotic modification (hours)	18.6 ± 6.2	52.4 ± 9.7	−33.8 (95% CI: −35.1 to −32.5)	<0.001
Diagnostic accuracy (%)	92.3	100 (reference)	–	–

Table 4 presents the distribution of genotypic resistance markers detected among the 410 bacterial isolates. The *blaCTX-M* gene was the most frequently detected resistance marker, identified in 134 isolates (32.7%), followed by *blaNDM* in 78 isolates (19.0%), *blaKPC* in 42 isolates (10.2%), and *mecA* in 54 isolates (13.2%). Other resistance genes were detected in 36 isolates (8.8%). Overall, 344 (83.9%) isolates had at least one of the reported genotypic resistance markers, whereas no resistance marker was detected among 66 (16.1%) isolates.

**Table 4:** Distribution of Genotypic Antimicrobial Resistance Markers Among Bacterial Isolates.

Resistance Gene	Frequency (n)	Percentage (%)
ESBL (*blaCTX-M*)	134	32.7
Carbapenemase (*blaNDM*)	78	19.0
Carbapenemase (*blaKPC*)	42	10.2
MRSA (*mecA*)	54	13.2
Others	36	8.8
No reported resistance marker	66	16.1

Moderate to substantial agreement was observed between genotypic resistance markers and phenotypic susceptibility findings, with Cohen’s kappa values ranging from 0.61 to 0.69. Concordance between blaCTX-M detection and phenotypic ESBL resistance was 84.6% (κ = 0.69), while carbapenem resistance demonstrated 81.2% concordance (κ = 0.61). MRSA isolates showed the highest percentage concordance, at 88.0%, with a Cohen’s kappa value of 0.62. Discordant cases underwent confirmatory phenotypic antimicrobial susceptibility testing and review by the antimicrobial stewardship team before final antimicrobial modification decisions were made. Concordance findings are summarized in Table 5.

**Table 5:** Concordance Between Genotypic Resistance Markers and Phenotypic Antimicrobial Susceptibility Results.

Resistance Type	Concordance (%)	Kappa Value
ESBL	84.6	0.69
Carbapenem Resistance	81.2	0.61
MRSA	88.0	0.62

Among patients managed during the study period, antimicrobial stewardship outcomes were compared between the pre-implementation period, during which conventional diagnostic workflows were used, and the post-implementation period, during which rapid diagnostic-supported workflows were available. Appropriate antibiotic use increased from 54.2% before implementation to 78.3% after implementation (absolute increase: 24.1%; p < 0.001). Broad-spectrum antibiotic utilization decreased from 72.4% to 49.3% (absolute reduction: 23.1%; p < 0.001). Similarly, antibiotic escalation rates declined from 38.9% to 21.7% (p < 0.001), whereas antibiotic de-escalation increased from 22.7% to 46.4% (p < 0.001). Stewardship-related outcomes are presented in Table 6.

**Table 6:** Changes in Antimicrobial Stewardship Indicators Before and After Implementation of Rapid Diagnostic-Supported Workflows.

Parameter	Before Rapid Diagnostics implementation (%)	After Rapid Diagnostics implementation (%)	Absolute Difference (%)	p-value
Appropriate antibiotic use	54.2	78.3	+24.1	<0.001
Broad-spectrum antibiotic use	72.4	49.3	−23.1	<0.001
Antibiotic escalation	38.9	21.7	−17.2	<0.001
Antibiotic de-escalation	22.7	46.4	+23.7	<0.001

Patients managed using rapid diagnostic-supported workflows had a shorter mean time to effective antimicrobial therapy than those managed using conventional diagnostic workflows (20.3 ± 7.1 hours versus 55.8 ± 11.2 hours; mean difference: −35.5 hours; 95% CI: −36.9 to −34.1; p < 0.001). Length of hospital stay was also significantly reduced in the rapid diagnostics group (8.6 ± 3.4 days versus 12.9 ± 4.8 days; mean difference: −4.3 days; 95% CI: −5.0 to −3.6; p < 0.001). Furthermore, patients managed using rapid diagnostic-supported workflows had lower odds of ICU admission (30.4% vs. 40.9%; OR 0.63; 95% CI: 0.42–0.95; p = 0.01) and in-hospital mortality (14.0% vs. 22.7%; OR 0.56; 95% CI: 0.33–0.93; p = 0.02) compared with those undergoing conventional testing. Clinical outcome comparisons are summarized in Table 7.

**Table 7:** Comparison of Clinical Outcomes Between Rapid Diagnostic-Supported and Conventional Diagnostic Workflows.

Outcome	Rapid Diagnostics	Conventional Testing	Effect Estimate	p-value
Time to effective therapy (hours)	20.3 ± 7.1	55.8 ± 11.2	−35.5 hours (95% CI: −36.9 to −34.1)	<0.001
Length of hospital stay (days)	8.6 ± 3.4	12.9 ± 4.8	−4.3 days (95% CI: −5.0 to −3.6)	<0.001
ICU admission (%)	30.4	40.9	OR 0.63 (95% CI: 0.42 to 0.95)	0.01
In-hospital mortality (%)	14.0	22.7	OR 0.56 (95% CI: 0.33 to 0.93)	0.02

The final machine learning model selected during internal validation demonstrated good discriminatory performance for predicting antimicrobial resistance. Overall model accuracy was 84.6%, with a sensitivity of 82.3% and specificity of 87.9%. The positive predictive value and negative predictive value were 90.3% and 78.4%, respectively. Receiver operating characteristic (ROC) analysis demonstrated an area under the curve (AUC) of 0.89, indicating excellent discriminatory ability. Calibration analysis demonstrated good agreement between predicted and observed probabilities, with a calibration slope of 1.04 and a calibration intercept of −0.02. The Hosmer–Lemeshow goodness-of-fit test showed no statistically significant evidence of poor calibration (χ² = 7.21, df = 8, p = 0.513). A probability threshold of 0.62 was prespecified from ROC analysis performed exclusively within the training dataset and was subsequently applied unchanged to the held-out validation dataset to classify patients as having a high predicted probability of antimicrobial resistance. This threshold was selected to balance sensitivity and specificity within the model development process. The performance metrics of the machine learning model are summarized in Table 8.

**Table 8:** Performance of the Machine Learning Model for Prediction of Antimicrobial Resistance.

Performance Metric	Value
Accuracy (%)	84.6
Sensitivity (%)	82.3
Specificity (%)	87.9
Positive Predictive Value (PPV) (%)	90.3
Negative Predictive Value (NPV) (%)	78.4
Area under the curve (AUC)	0.89
Probability threshold (Cutoff)	0.62

Multivariable logistic regression analysis identified several independent factors associated with in-hospital mortality (Table 9). Patients aged >60 years had higher adjusted odds of mortality than younger patients (AOR 2.56; 95% CI: 1.50–4.37; p < 0.001), while ICU admission was also independently associated with higher mortality (AOR 2.81; 95% CI: 1.66–4.77; p < 0.001). The presence of antimicrobial resistance was associated with increased adjusted odds of mortality (AOR 1.95; 95% CI: 1.12–3.41; p = 0.019). Delayed initiation of effective therapy beyond 48 hours was not independently associated with mortality (AOR 1.02; 95% CI: 0.47–2.22; p = 0.955). After adjustment for the variables included in the model, rapid diagnostic use was not statistically significantly associated with mortality (AOR 0.59; 95% CI: 0.27–1.28; p = 0.184), despite the lower unadjusted mortality observed in the rapid diagnostic group.

**Table 9:** Multivariable Logistic Regression Analysis of Factors Associated with In-Hospital Mortality.

Variable	AOR	95% CI	p-value
Age > 60 years	2.56	1.50-4.37	<0.001
ICU Admission	2.81	1.66-4.77	<0.001
AMR presence	1.95	1.12-3.41	0.019
Delayed therapy (>48 hrs)	1.02	0.47-2.22	0.955
Rapid diagnostics use	0.59	0.27-1.28	0.184

Comparative cost analysis demonstrated that although rapid diagnostic testing incurred higher direct diagnostic costs compared with conventional methods (PKR 33,600 versus PKR 12,600), overall hospitalization costs were substantially lower due to shorter hospital stay and earlier optimization of antimicrobial therapy. Mean total cost per patient was PKR 308,000 in the rapid diagnostics group compared with PKR 418,600 in the conventional testing group, corresponding to estimated net savings of approximately PKR 110,600 per patient. Findings are summarized in Table 10.

**Table 10:** Comparison of Diagnostic, Hospitalization, and Total Costs Between Conventional and Rapid Diagnostic-Supported Workflows.

Parameter	Conventional (PKR)	Rapid Diagnostics (PKR)	Difference (PKR)
Diagnostic cost	12,600	33,600	+21,000
Hospitalization cost	406,000	274,400	−131,600
Total cost	418,600	308,000	−110,600
Estimated net cost difference per patient	–	–	110,600

Table 11 presents the results of a multivariable logistic regression model examining the independent associations of the three diagnostic and predictive components with appropriate antibiotic therapy. The model additionally included prespecified clinical covariates selected a priori based on clinical relevance and previously published evidence. Rapid diagnostic testing, genotypic resistance results, and machine learning prediction outputs were entered simultaneously into the multivariable model, together with prespecified clinical covariates, to estimate their independent associations with appropriate antibiotic therapy. Rapid diagnostic testing was associated with higher odds of appropriate antimicrobial selection (OR 3.22; 95% CI: 2.05–5.07; p < 0.001). Machine learning prediction outputs were independently associated with higher odds of appropriate antibiotic therapy (OR 2.23; 95% CI: 1.22–4.08; p = 0.001), while incorporation of genotypic resistance results was associated with higher odds of appropriate antibiotic therapy (OR 1.85; 95% CI: 1.01–3.38; p = 0.02). These findings provide exploratory estimates of the independent associations of each component but do not establish causal effects or quantify the incremental benefit of sequentially adding each component to the diagnostic workflow. Collectively, the findings suggest that integration of rapid diagnostics, genotypic resistance profiling, and machine learning-assisted prediction may support optimization of antimicrobial decision-making within institutional antimicrobial stewardship workflows.

**Table 11:** Multivariable Logistic Regression Analysis of Factors Associated with Appropriate Antibiotic Therapy.

Variable	AOR	95% CI	p-value
Rapid diagnostics	3.22	2.05-5.07	<0.001
AI prediction	2.23	1.22-4.08	0.001
Genotypic results	1.85	1.01-3.38	0.02

## Discussion

This study demonstrated that integration of rapid diagnostic testing, genotypic resistance profiling, and machine learning-assisted prediction was associated with significant improvements in antimicrobial stewardship metrics and clinical outcomes among patients with suspected bacterial infections. A primary finding was the significant reduction in time to pathogen identification and susceptibility reporting, which facilitated earlier initiation of effective antimicrobial therapy. These results are consistent with prior studies indicating that rapid diagnostic platforms may reduce the time to appropriate therapy by 24–48 hours and may facilitate earlier clinical decision-making [15,16].

An important finding of the present study was the significant improvement in appropriate antibiotic utilization following implementation of rapid diagnostics. Appropriate antibiotic therapy increased from 54.2% to 78.3%, while broad-spectrum antibiotic use declined significantly. Similarly, antibiotic de-escalation rates increased substantially after integration of rapid diagnostic tools. These findings support the growing evidence that antimicrobial stewardship programs (ASPs) benefit considerably from timely microbiological information, enabling clinicians to minimize unnecessary broad-spectrum antibiotic exposure while improving therapeutic precision [17,18].

The study also demonstrated that patients managed using rapid diagnostic-supported workflows had shorter hospital stays and lower unadjusted in-hospital mortality. Patients managed using the integrated diagnostic approach experienced earlier initiation of effective antimicrobial therapy and a shorter duration of hospitalization compared with those managed using conventional diagnostic workflows alone. Similarly, ICU admission and in-hospital mortality were lower among patients managed using rapid diagnostic-supported workflows (ICU admission: 30.4% vs. 40.9%, OR 0.63, 95% CI 0.42–0.95; mortality: 14.0% vs. 22.7%, OR 0.56, 95% CI 0.33–0.93). These findings are consistent with previous evidence demonstrating that rapid diagnostic testing integrated with antimicrobial stewardship can facilitate timely optimal therapy and is associated with reductions in length of hospital stay and mortality among patients with bloodstream infections [17,19]. However, given the observational nature of the present study, these findings should be interpreted as associations rather than definitive causal effects.

Substantial concordance was observed between genotypic resistance markers and phenotypic antimicrobial susceptibility testing, with Cohen’s kappa values ranging from 0.61 to 0.69, indicating moderate to substantial agreement. These findings are broadly consistent with previous evidence demonstrating meaningful concordance between genotypic resistance determinants and phenotypic antimicrobial susceptibility profiles among ESBL-producing *E. coli* and carbapenem-resistant *K. pneumoniae* [20,21]. Nevertheless, discordant cases were identified and were managed through confirmatory phenotypic susceptibility testing prior to final antimicrobial modification decisions. In situations where genotypic and phenotypic findings differed, clinicians prioritized conventional phenotypic AST results in consultation with the antimicrobial stewardship and microbiology teams. This approach was adopted to reduce the risk of inappropriate therapy due to incomplete molecular resistance coverage or variable gene expression.

The machine learning prediction model demonstrated strong discriminatory performance, with an AUC of 0.89, sensitivity of 82.3%, specificity of 87.9%, and overall accuracy of 84.6%. The positive predictive value and negative predictive value were 90.3% and 78.4%, respectively. These findings are broadly consistent with the growing body of evidence supporting the potential utility of machine learning approaches for antimicrobial resistance prediction and clinical decision support, although reported model performance varies substantially across studies and clinical settings [22,23]. Importantly, the present model should be considered an internally validated derivation model rather than a clinically deployable tool. Although the model demonstrated good discrimination and acceptable calibration, external validation across multiple healthcare settings and patient populations remains necessary before routine clinical implementation can be recommended. The integration of machine learning predictions alongside rapid diagnostics and genotypic profiling may nonetheless provide additional support for early risk stratification and antibiotic selection, particularly in healthcare systems with limited infectious disease expertise.

Economic analysis demonstrated that although rapid diagnostic testing was associated with higher upfront diagnostic costs, the observed mean total hospitalization cost was lower, primarily attributable to reduced hospital length of stay and more efficient antimicrobial optimization. These findings are consistent with previous evidence indicating that rapid diagnostic testing, particularly when integrated with antimicrobial stewardship interventions, may reduce healthcare costs and be economically favorable when appropriately implemented. In low- and middle-income countries, where antimicrobial resistance and prolonged hospitalization impose a considerable economic burden on healthcare systems, integrated diagnostic stewardship approaches may therefore offer important economic advantages [19,24]. Furthermore, previous studies have reported that effective antibiotic de-escalation strategies are associated with shorter lengths of stay and lower healthcare costs, supporting the plausibility of the cost reductions observed in the present analysis.

This study has several important limitations. First, the single-center design may limit generalizability to other institutions and healthcare systems. Second, although multivariable regression was performed, residual confounding inherent to observational studies cannot be completely excluded. Third, the machine learning model underwent only internal validation and therefore requires prospective external validation prior to broader clinical adoption. Fourth, molecular resistance profiling was limited to selected resistance genes and may not fully capture the complexity of antimicrobial resistance mechanisms. Finally, exclusion of polymicrobial infections may reduce applicability to some real-world clinical scenarios involving mixed infections.

Despite these limitations, the study has notable strengths, including prospective data collection, integration of multiple diagnostic modalities within routine clinical workflows, inclusion of clinically relevant stewardship outcomes, and incorporation of machine learning-assisted prediction in a resource-limited setting. Collectively, the findings support the potential value of integrated rapid diagnostics, molecular resistance profiling, and machine learning-assisted stewardship approaches in optimizing antimicrobial therapy and strengthening antimicrobial stewardship practices. Future multicenter studies incorporating external model validation, expanded molecular testing panels, and real-time clinical decision-support integration are warranted to further establish the effectiveness and scalability of these approaches.

## Conclusion

This prospective observational study found that an integrated diagnostic stewardship approach combining rapid diagnostic testing, genotypic resistance profiling, and machine learning-assisted prediction was associated with improved antimicrobial stewardship practices and favorable clinical outcomes among patients with suspected bacterial infections. The integrated approach was associated with shorter times to effective therapy, higher rates of appropriate antibiotic selection, lower broad-spectrum antibiotic utilization, higher rates of antibiotic de-escalation, and shorter hospital stays. Furthermore, the machine learning prediction model demonstrated strong discriminatory performance and may provide additional support for early antimicrobial decision-making when used alongside conventional microbiological assessment and stewardship review. Substantial concordance between genotypic and phenotypic resistance patterns further supports the complementary role of molecular diagnostics in antimicrobial management. Although rapid diagnostics were associated with higher initial testing costs, the observed reductions in total hospitalization costs suggest potential economic advantages, particularly in resource-limited healthcare settings with high antimicrobial resistance burdens. However, given the observational single-center design and internally validated nature of the machine learning model, further multicenter prospective studies with external validation are required before widespread clinical implementation can be recommended. Overall, the findings support the potential role of integrated rapid diagnostics, molecular resistance profiling, and machine learning-assisted stewardship strategies in strengthening antimicrobial stewardship and optimizing patient care in low- and middle-income healthcare systems.

### Highlights

- Integrated rapid diagnostics, genotypic resistance profiling, and AI prediction significantly optimize antimicrobial stewardship.
- Rapid testing accelerated pathogen identification and initiation of effective therapy, reducing hospital stay and mortality.
- AI-based models achieved high predictive performance (AUC = 0.89) for targeted antibiotic therapy.
- Substantial genotypic–phenotypic concordance validates the clinical relevance of molecular diagnostics alongside conventional AST.
- The combined approach was associated with lower overall hospitalization costs and may offer a scalable strategy to combat antimicrobial resistance in resource-limited healthcare settings.

## Abbreviations

AMR: Antimicrobial Resistance
AST: Antibiotic Susceptibility Testing
AI: Artificial Intelligence
AUC: Area Under the Curve
CI: Confidence Interval
CKD: Chronic Kidney Disease
ESBL: Extended-Spectrum Beta-Lactamase
ICU: Intensive Care Unit
MRSA: Methicillin-Resistant *Staphylococcus aureus*
NDM: New Delhi Metallo-beta-lactamase
KPC: *Klebsiella pneumoniae Carbapenemase*
PPV: Positive Predictive Value
NPV: Negative Predictive Value
ROC: Receiver Operating Characteristic
UTI: Urinary Tract Infection
PKR: Pakistani Rupees
OR: Odds Ratio
AOR: Adjusted Odds Ratio
SD: Standard Deviation
CLSI: Clinical and Laboratory Standards Institute.
